# Supplementary material for: Optical interferometry based micropipette aspiration provides real-time sub-nanometer spatial resolution
Source: Commun Biol. 2021 May 21;4:610. doi: 10.1038/s42003-021-02121-1 (PMC8140111; doi:10.1038/s42003-021-02121-1)
Supplement: Supplementary file 2 — Supplementary Information [file 42003_2021_2121_MOESM2_ESM.pdf]

Supplementary Information to: Optical Interferometry Based Micropipette Aspiration Provides Real-time Sub-nanometer Spatial Resolution

Massimiliano Berardi[1,2,*], Kevin Bielawski[2], Niek Rijnveld[2], Grzegorz Gruca[2], Hilde Aardema[3], Leni van Tol[3], Gijs Wuite[1], and B. Imran Akca[1,**]

[1]LaserLab, Department of Physics and Astronomy, VU University, De Boelelaan 1081, 1081 HV, Amsterdam, The Netherlands
[2]Optics11, De Boelelaan 1081, 1081 HV, Amsterdam, The Netherlands
[3]Department of Farm Animal Health, Faculty of Veterinary medicine, Utrecht University, Yalelaan 7, 3584 Cl Utrecht, the Netherlands
[*]massimiliano.berardi@optics11.com
[**]b.i.avci@vu.nl

April 8, 2021

1 Supplementary Note 1: MPA Probe and Testing Setup

Since we do not own a microforge, we had to develop laser-ablation based method to manufacture micropipettes. We used an optical profiler to characterize the surface of the micropipettes. We found Rq values oscillating between 100 and 150 nm, which is higher than what one would obtain with fire polish. However, since we are using relatively large capillaries coupled with limited deformation, we can assume the effect of the rough boundary, compared to a smooth support, to be limited. The different contact may change the boundary condition of sliding that the theory requires. For instance, if the membrane was clamped at the nozzle, we would overestimate the mechanical properties We modeled the Zona Pellucida assuming the boundary effects to be minimum, if present, as we are measuring very small radial displacements at the center of the nozzle that is several microns away from the contact area.

2 Supplementary Note 2: Optical Characterization

As mentioned in the main text, the use of interferometry allows to characterize the samples optically. An optical cavity of refractive index n_i can be seen it if it is larger than the axial resolution of system, which depends on the wavelength choice and the bandwidth of the source[1]. In our case, the minimum detectable cavity is about 22 μm. An example is given Figure 1-e in the main text. A Polystyrene microbead (160 μm certified size standard, Duke Scientific) is captured by applying a low negative pressure. The corresponding Fourier-transformed interferogram shows four peaks; from left to right (a) pressure sensor, (b) bead front to back, (c) fiber to bead front, (d) compound cavity (fiber to bead and bead front to back). Knowing the RI $n_c \approx 1.58$[2], we can estimate the sample size to be b$\approx$158 μm (either from the first peak or the fourth), which corresponds with the brightfield measurement and is contained within the declared distribution (160$\pm$3.5 μm). On top of this, the signal retrieval is also affected by the geometry of the sample, and the incidence angle of light, which can affect how certain cavities are visible. An example is given in the Supplementary

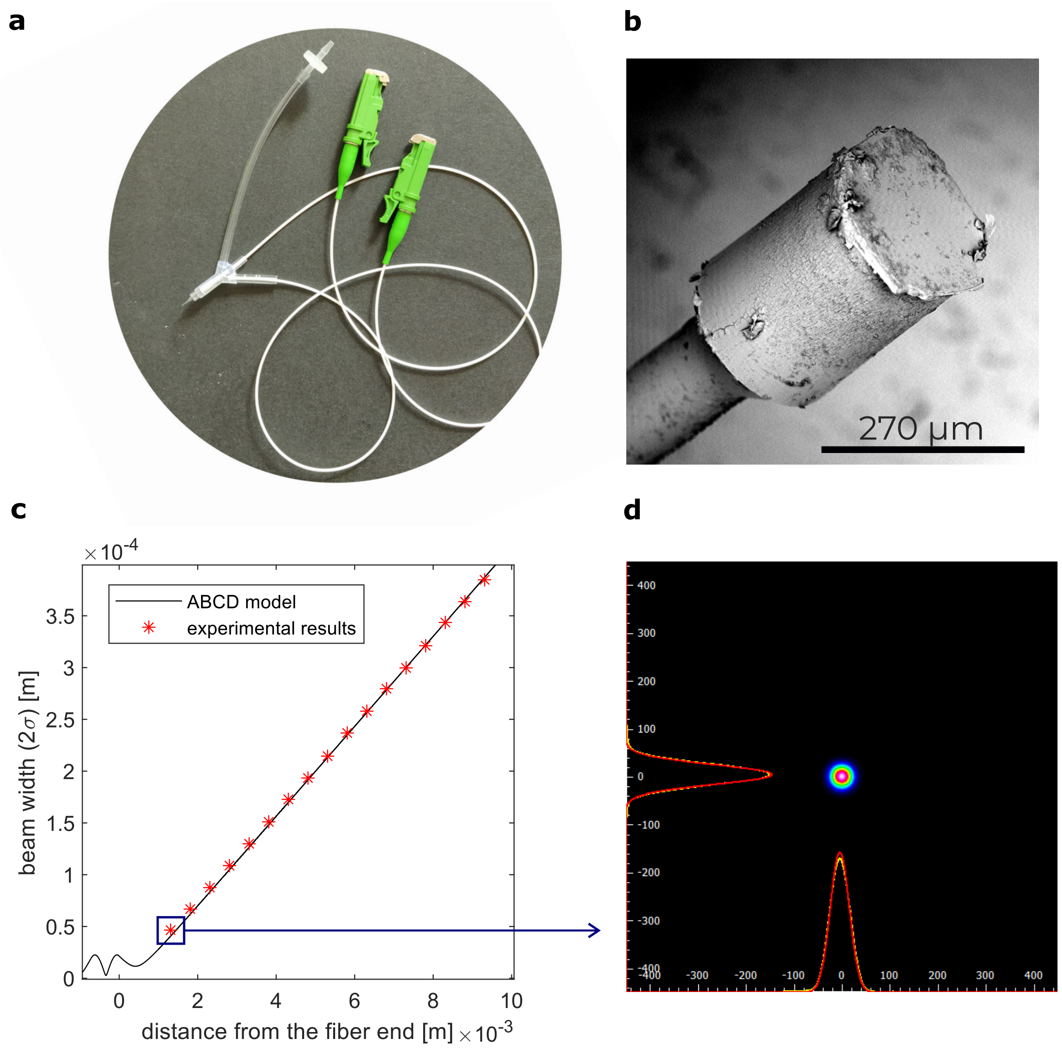

Supplementary Figure 1: Assembled probe and highlight of its sensing components. (a) Picture of the completed prove. (b) SEM image of the pressure sensor mounted on a single mode fiber. (c) Predicted beam profile (using the ABCD matrix method) against the experimental measurement. Note that the prediction for negative distance value refers to the behavior of light whilst traversing the coreless fiber and the GRIN lens. (d) 2D reconstruction of the beam emerging from the uGRIN lens, approximately 1.8 mm from the fiber end facet. In yellow, the measured profile, and in red the gaussian fit. Dimensions in microns.

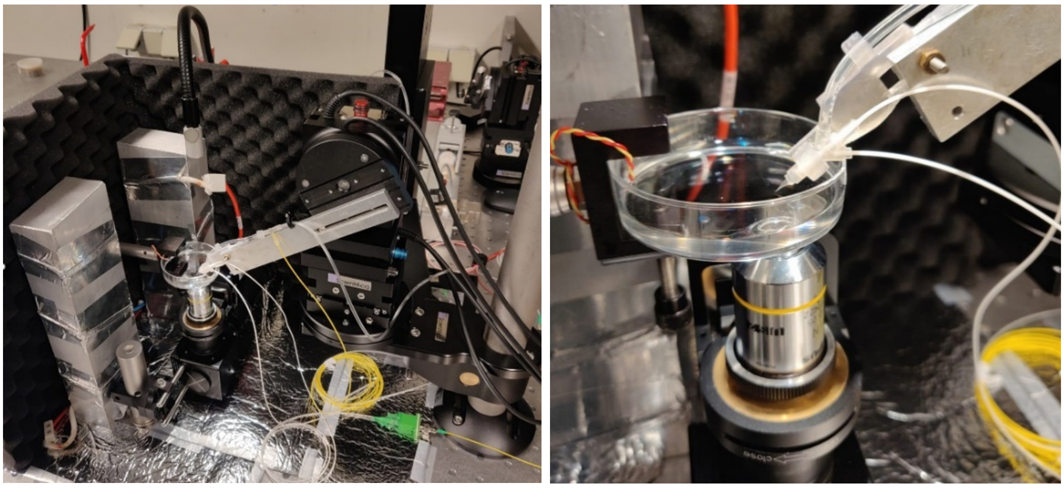

Supplementary Figure 2: The setup used to validate the probe, with the front portion of the isolation box removed. The blocks of aluminium visible on the left picture were wrapped in wire and used as heating elements. On the right it is possible to see the probe, mounted on a motorized arm, immersed in a Petri dish, and the thermocouple used for temperature control (braided yellow/red cable on the left).

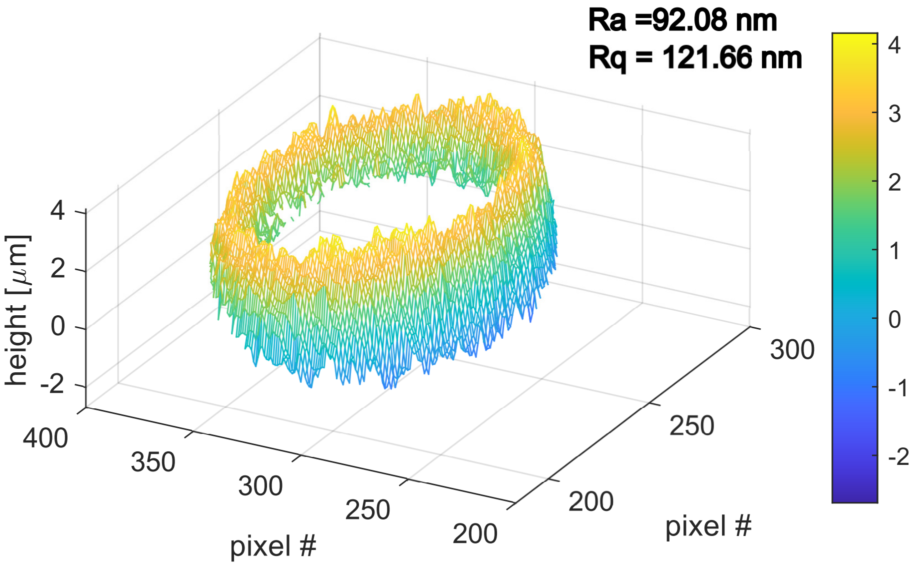

Supplementary Figure 3: Example of the surface of a capillary tip manufactured via pulling followed by laser ablation. The profile was obtained with an optical scanning profiler (Veeco WYKO NT9100).

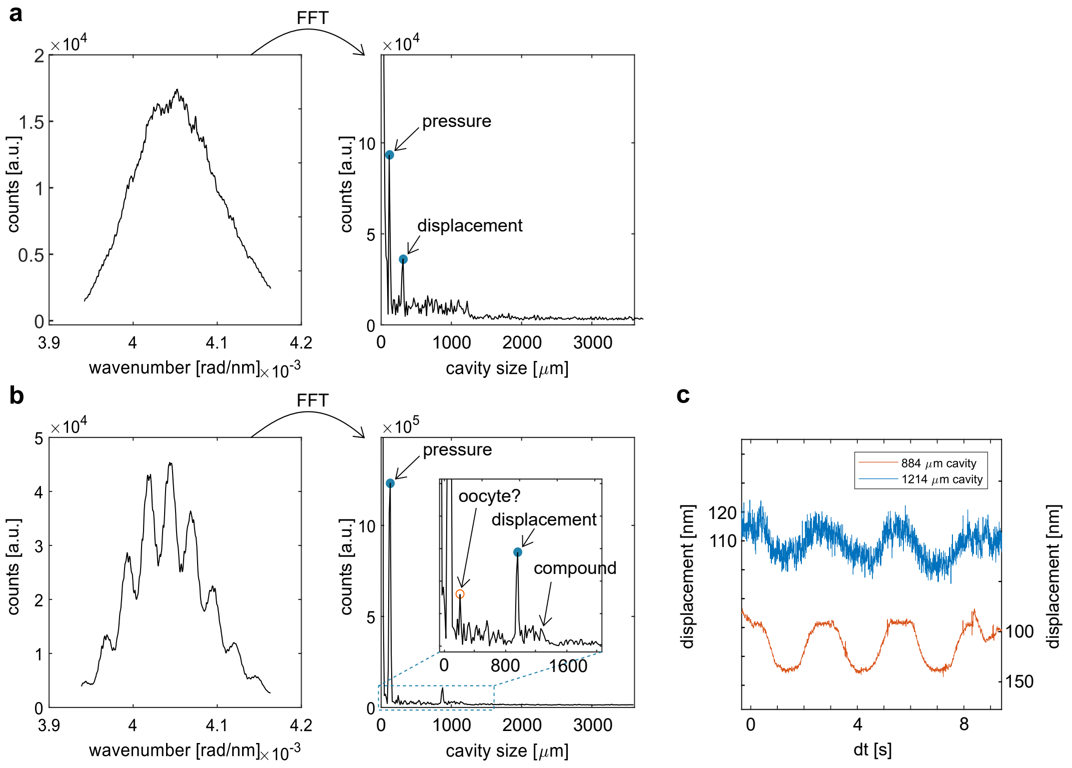

Supplementary Figure 4: Examples of interferograms and cavity spaces highlighting the pressure sensor, the fiber to sample distance and a possible sample related peak for (a) an alginate hydrogel microbead and (b) a mature bovine oocyte. (c) shows a detail of the demodulated phases for the displacement (880 μm) and compound (1214 μm) peak.

Movie 2: whilst the configuration is remarkably similar to what shown in Figure 1-e, the only peaks that can be recognized are the fiber-to-sample and the compound one. The alginate microbead (Supplementary Figure 4-a), owing to its high water content, provided a very weak signal overall, and we were able to identify only the fiber to sample cavity, aside from the one defined by the pressure sensor. Similarly, when measuring the flying fish egg, we were only able to retrieve the first cavity signal. In this case, it is probably due to its large size couple with strong signal absorption characteristics. In contrast, when testing the bovine oocyte, we were able to observe an additional peak. Its position ($\approx$334 μm) divided by the oocyte diameter measured in brightfield ($\approx$160 μm) yields a refractive index of 2.12, and its phase demodulation appears flat. A previous study[3] reported refractive indices for bovine oocytes cytoplasm and Zona Pellucida in the range 1.88$\pm$0.05 and 2.32$\pm$0.022 respectively, which are close to our measurement. Additionally, by demodulating the phase at 1215 μm (i.e. the expected location of the compound peak), we retrieved the same signal from the fiber-to-sample cavity, albeit with a significantly lower signal-to-noise ratio (see Supplementary Figure 4-c). The second peak is not a harmonic of the fiber to sample cavity nor of the pressure sensor, so we can assume it is in fact a signal coming from the oocyte. This seems to support the hypothesis of the peak describing the average optical cavity the sample, as the Zona Pellucida is only 10 μm thick, hence would not be detectable as an isolated peak.

3 Supplementary Note 3: Testing Results

3.1 Validation Against Video Tracking

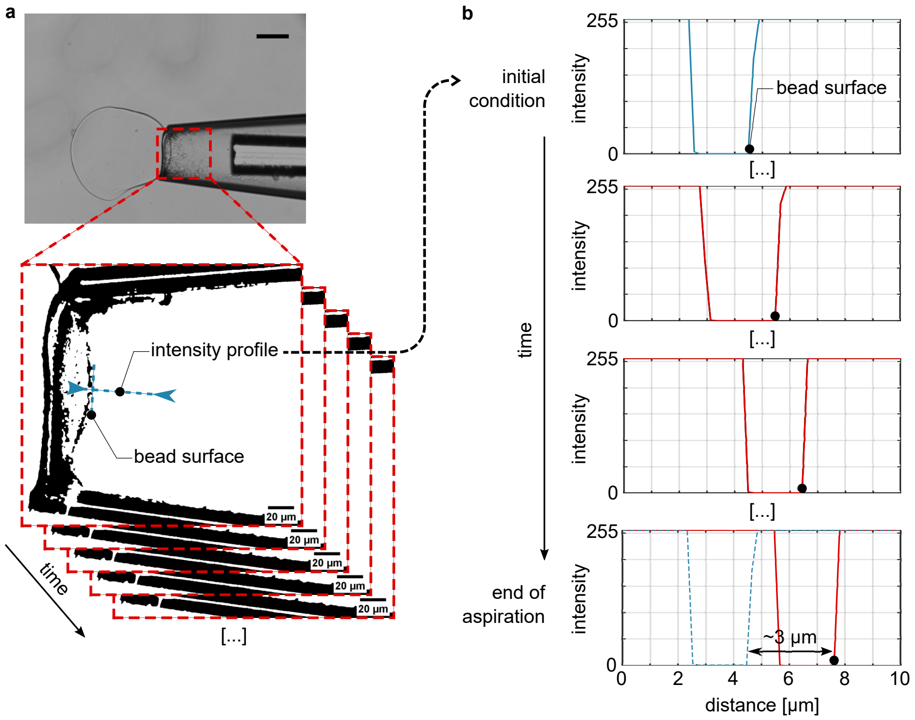

Supplementary Figure 5: Video tracking method for monitoring the aspirated length of the microbead in the capillary. (a) During an experiment, we recorded a timelapse of the capillary tip (7 fps, 50x objective). We then took the image stack and applied a binary filter, to simplify the membrane tracking. Using ImageJ, we defined a line crossing the bead membrane, located at the center of the nozzle and perpendicular to it. We used it to plot the pixel intensity along its length for each frame. The series of plots (b) shows the evolution of the intensity profile over time: as the bead is drawn into the pipette by the suction pressure, the dark region moves to the right. We defined the right side of the intensity drop as the bead surface. In the bottom right plot, the initial and final condition have bee plotted together, to show the aspirated length variation.

3.2 DMA on Flying Fish Roe

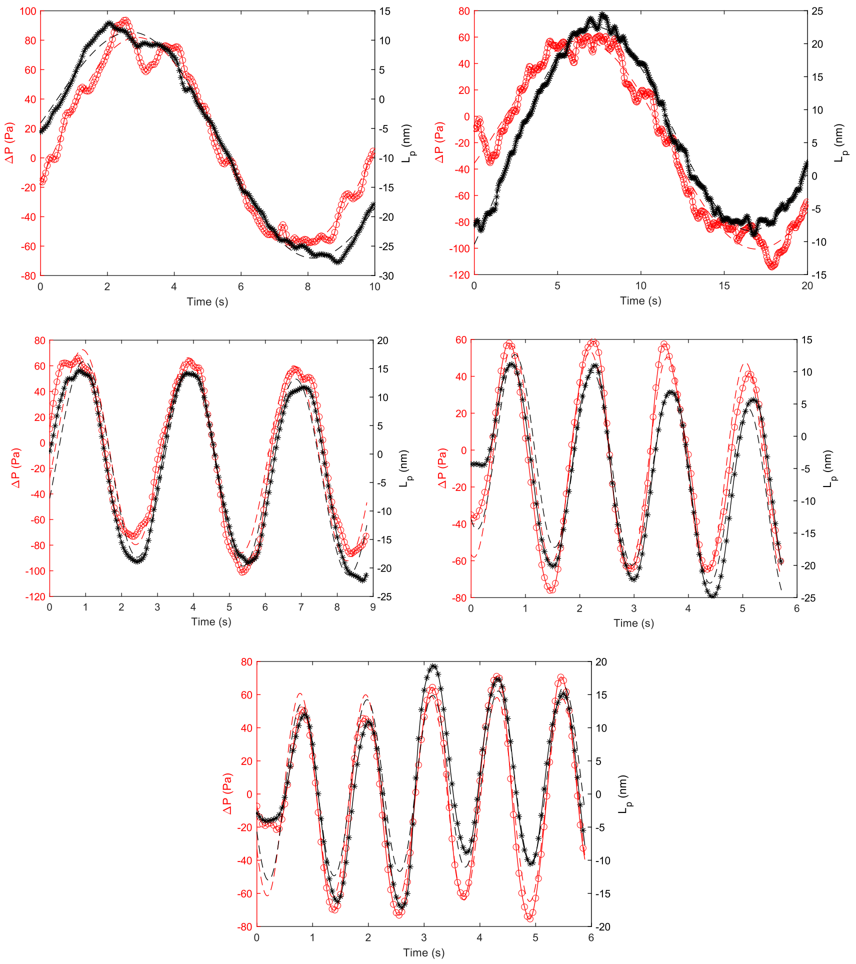

Supplementary Figure 6: Examples of a DMA experiment on the flying fish roe. Fit (dashed lines) vs experimental data ('*' for aspirated length, 'o' for pressure) at each frequency.

frequency [Hz]	0.05	0.1	0.35	0.75	1
E' [kPa]	338±75	347±43	396±54	411±89	445±91
E" [kPa]	46±73	28±33	66±33	87±61	77±74
$\tan\delta$	0.14±0.17	0.08±0.10	0.17±0.09	0.22±0.12	0.17±0.11

Supplementary Table 1: Results of the DMA analysis of flying fish roe(N=10).

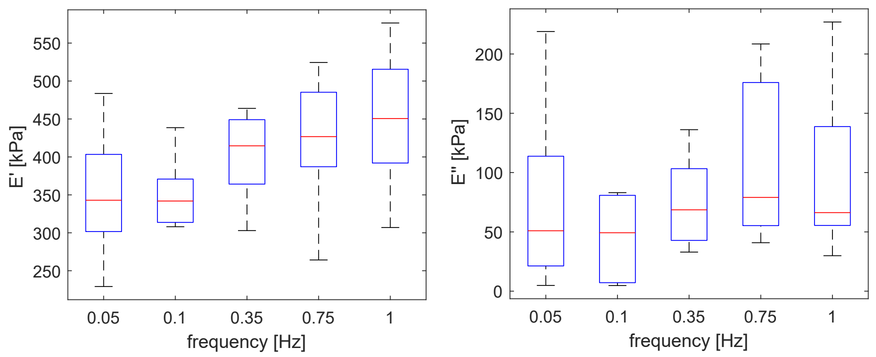

Supplementary Figure 7: Boxplot of DMA analysis of flying fish roe (N=10

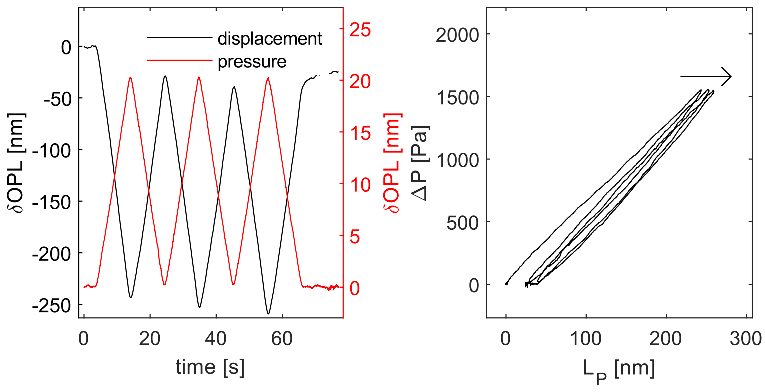

Supplementary Figure 8: Progressive loading-unloading of a fish roe shows progressive softening of the outer shell. The significant increase in resolution, compared to the standard method, allows observing the effect of load preconditioning a sample. As the fish egg is subject to repeated loading/unloading, its mechanical response changed due to its viscous characteristic.

3.3 DMA on Bovine Oocytes

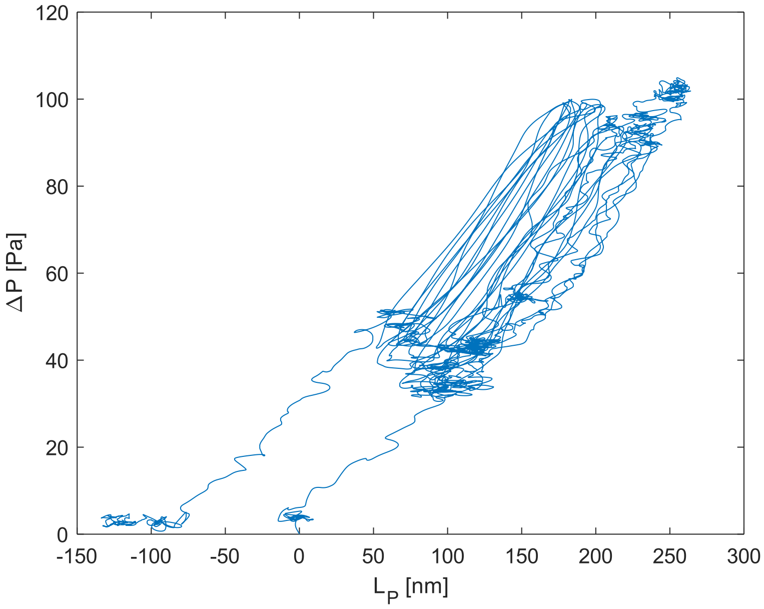

Supplementary Figure 9: Example of the dP vs Lp behavior during a DMA of an oocyte.The test begins with the capture and initial aspiration as shown at the bottom left of the plot. The test starts by applying a prestress on the Zona Pellucida. Once the target preload is reached, the pressure oscillations start. The Zona Pellucida has a strong viscous characteristic and during the test creep deformation occurs, on top of the dynamic one. This can be seen in the looping part of the curve, where the curves, whist confined between 50 and 100 Pa, tend to shift to the right, which indicates the Zona Pellucida is creeping in the pipette.

frequency [Hz]	0.05	0.1	0.35	0.75	1
E' [kPa]	39.2±6.3	47.7±10.9	54.1±13.3	59.9±17.3	61.9±19.3
E" [kPa]	6.0±4.9	8.8±5.0	11.1±5.3	13.4±6.3	13.7±7.1
$\tan\delta$	0.1±0.11	0.18±0.7	0.20±0.05	0.22±0.05	0.21±0.05

Supplementary Table 2: Results of the DMA analysis of bovine oocytes (N=10).

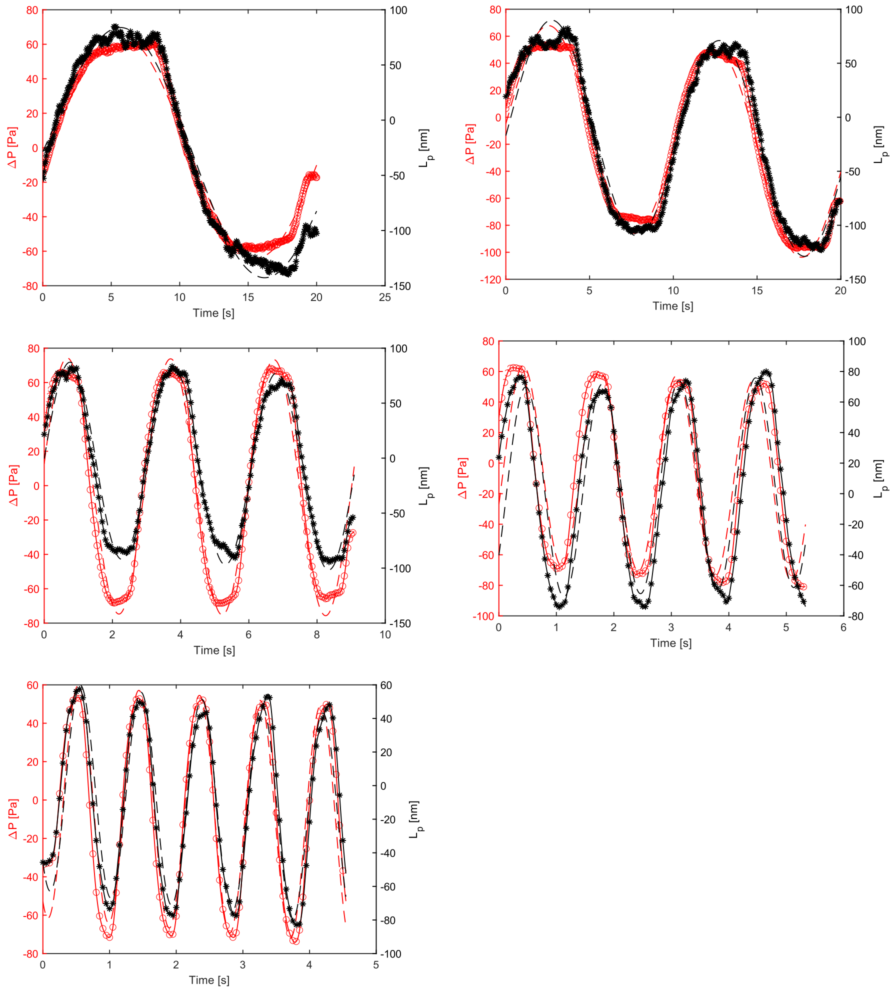

Supplementary Figure 10: Examples of a DMA experiment on a bovine oocyte. Fit (dashed lines) vs experimental data ('*' for aspirated length, 'o' for pressure) at each frequency.

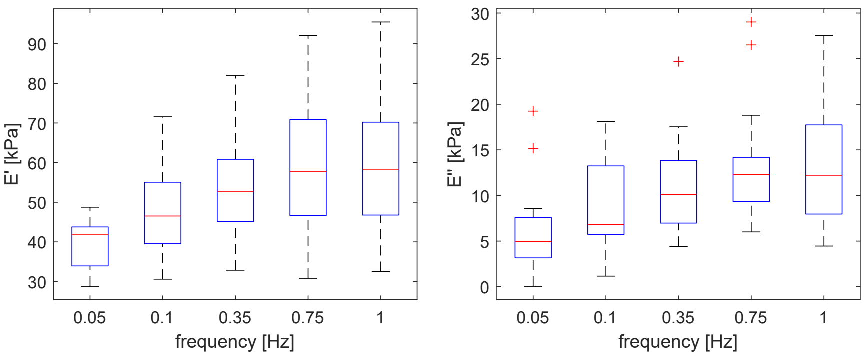

Supplementary Figure 11: Boxplot of the DMA results on the 10 tested oocytes.

Supplementary References

1. Ikeda, N. & Lam, S. Optical Coherence Tomography. in Interventions in Pulmonary Medicine 231–236 (Springer New York, 2013). doi:10.1007/978-1-4614-6009-1-15.

2. Ma, X., Lu, J. Q., Brock, R. S., Jacobs, K. M., Yang, P., & Hu, X. H. Determination of complex refractive index of polystyrene microspheres from 370 to 1610 nm. Phys. Med. Biol. 48(24), 4165–4172 (2003).

3. Wacogne, Bruno et al. Microsensors and image processing for single oocyte qualification: toward multiparametric determination of the best time for fertilization, Laser Physics Letters, IOP Publishing, 10. (2013).
