# Supplementary material for: Optical interferometry based micropipette aspiration provides real-time sub-nanometer spatial resolution
Source: Commun Biol. 2021 May 21;4:610. doi: 10.1038/s42003-021-02121-1 (PMC8140111; doi:10.1038/s42003-021-02121-1)
Supplement: Supplementary file 3 — Description of Additional Supplementary Files [file 42003_2021_2121_MOESM3_ESM.pdf]

Description of Additional Supplementary Files

File name: Supplementary Movie 1

Description: Capture of a glass bead, approximately 100 um in diameter. We show both the video feed from the microscope and the interferogram and its Fourier transform, highlighting the formation of optical cavities that occur when capturing the bead.

File name: Supplementary Movie 2

Description: Release of a PS bead. In this case, we show how a different nozzle diameter and fiber to sample distance provide a different interferogram, where we cannot see the peak belonging to the bead alone, but only the one compounded with the first cavity. Additionally, we show the real time phase demodulation for the pressure sensor and the aspirated length, and their changes when a small positive pressure is applied at the nozzle.
