# Supplementary material for: Optical interferometry based micropipette aspiration provides real-time sub-nanometer spatial resolution
Source: Commun Biol. 2021 May 21;4:610. doi: 10.1038/s42003-021-02121-1 (PMC8140111; doi:10.1038/s42003-021-02121-1)
Supplement: Supplementary file 6 — Reporting Summary [file 42003_2021_2121_MOESM6_ESM.pdf]

nature research

Corresponding author(s): Massimiliano Berardi and B. Imran Avci

Last updated by author(s): 04 December 2020

Reporting Summary

Nature Research wishes to improve the reproducibility of the work that we publish. This form provides structure for consistency and transparency in reporting. For further information on Nature Research policies, see our Editorial Policies and the Editorial Policy Checklist.

Statistics

For all statistical analyses, confirm that the following items are present in the figure legend, table legend, main text, or Methods section.

n/a	Confirmed	
☐	☒	The exact sample size (n) for each experimental group/condition, given as a discrete number and unit of measurement
☐	☒	A statement on whether measurements were taken from distinct samples or whether the same sample was measured repeatedly
☐	☒	The statistical test(s) used AND whether they are one- or two-sided<br>*Only common tests should be described solely by name; describe more complex techniques in the Methods section.*
☒	☐	A description of all covariates tested
☐	☒	A description of any assumptions or corrections, such as tests of normality and adjustment for multiple comparisons
☐	☒	A full description of the statistical parameters including central tendency (e.g. means) or other basic estimates (e.g. regression coefficient) AND variation (e.g. standard deviation) or associated estimates of uncertainty (e.g. confidence intervals)
☒	☐	For null hypothesis testing, the test statistic (e.g. F, t, r) with confidence intervals, effect sizes, degrees of freedom and P value noted<br>*Give P values as exact values whenever suitable.*
☒	☐	For Bayesian analysis, information on the choice of priors and Markov chain Monte Carlo settings
☒	☐	For hierarchical and complex designs, identification of the appropriate level for tests and full reporting of outcomes
☒	☐	Estimates of effect sizes (e.g. Cohen's d, Pearson's r), indicating how they were calculated

Our web collection on statistics for biologists contains articles on many of the points above.

Software and code

Policy information about availability of computer code

Data collection	DeltaSens (Optics 11), Matlab (2019), custom code for readout
Data analysis	A custom made code using Matlab for ray optics part. DeltaSens to read interferometric data.

For manuscripts utilizing custom algorithms or software that are central to the research but not yet described in published literature, software must be made available to editors and reviewers. We strongly encourage code deposition in a community repository (e.g. GitHub). See the Nature Research guidelines for submitting code & software for further information.

Data

Policy information about availability of data

All manuscripts must include a data availability statement. This statement should provide the following information, where applicable:
- Accession codes, unique identifiers, or web links for publicly available datasets
- A list of figures that have associated raw data
- A description of any restrictions on data availability

We provided this statement.

Field-specific reporting

Life sciences study design

All studies must disclose on these points even when the disclosure is negative.

Sample size	For the biological samples, the sample number was limited by the amount of available samples provided by our collaborators.
Data exclusions	No data was excluded.
Replication	All measurements were repeated several times at different times to confirm the data reproducibility.
Randomization	We did not randomize the samples. We followed a path to be able to first prove the principle on non-biological samples, and later on biological samples.
Blinding	Yes, they were blinded.

Reporting for specific materials, systems and methods

We require information from authors about some types of materials, experimental systems and methods used in many studies. Here, indicate whether each material, system or method listed is relevant to your study. If you are not sure if a list item applies to your research, read the appropriate section before selecting a response.

Materials & experimental systems

n/a	Involved in the study
☒	☐ Antibodies
☒	☐ Eukaryotic cell lines
☒	☐ Palaeontology and archaeology
☐	☒ Animals and other organisms
☒	☐ Human research participants
☒	☐ Clinical data
☒	☐ Dual use research of concern

Methods

n/a	Involved in the study
☒	☐ ChIP-seq
☒	☐ Flow cytometry
☒	☐ MRI-based neuroimaging

Animals and other organisms

Policy information about studies involving animals; ARRIVE guidelines recommended for reporting animal research

Laboratory animals	We did not use the animal itself, we used the oocytes taken from already slaughtered animals.
Wild animals	This study did not involve any wild animal.
Field-collected samples	Bovine oocytes were collected from a cow uterus, which was provided by a local butcher. The collection procedure was done by our collaborators from Utrech University Veterinary Medicine.
Ethics oversight	No ethical approval was needed for this.

Note that full information on the approval of the study protocol must also be provided in the manuscript.
